# Stepwise−Process−Controlled Ligand Management Strategy for Efficient and Stable Perovskite Quantum Dot Solar Cells

**DOI:** 10.3390/nano13233032

**Published:** 2023-11-27

**Authors:** Jinfei Dai, Wei Guo, Jie Xu, Ruoyao Xu, Jun Xi, Hua Dong, Zhaoxin Wu

**Affiliations:** 1 Key Laboratory for Physical Electronics and Devices of the Ministry of Education, School of Electronic Science and Engineering, Xi’an Jiaotong University, Xi’an 710049, China; 2 Shaanxi Key Lab of Information Photonic Technique, School of Electronic Science and Engineering, Xi’an Jiaotong University, Xi’an 710049, China; 3 College of Science, Xi’an University of Architecture and Technology, Xi’an 710055, China; jiexu@xauat.edu.cn

**Keywords:** CsPbI_3_ quantum dot, ligand exchange, device stability, perovskite solar cell

## Abstract

CsPbI_3_ perovskite quantum dots (QDs) have attracted much attention in the field of solar cells because of their excellent photovoltaic properties. Conventional modification of long−chain insulating ligands can ensure good dispersion and film−forming stability of QDs, but the limitations of their low defect passivation ability and poor charge transport ability will make them fail to achieve high efficiency in the corresponding solar cell devices. In this study, by introducing “Benzylphosphonic acid” short−chain ligands to the surface of CsPbI_3_ QDs, the ligands were re−administered on the surface during the preparation of the CsPbI_3_ QDs as well as during the film−forming process. The strong coordination ability of Benzenephosphonic acid can effectively passivate defects on the surface of CsPbI_3_ QDs and inhibit non−radiative recombination and phase transition. Meanwhile, this short−chain ligand can effectively promote the charge exchange between adjacent QDs and improve the electrical transport properties of the film. The efficiency of the Benzylphosphonic acid−modified CsPbI_3_ QDs solar cell reaches 13.91% compared to the unmodified device (PCE of 11.4%). The storage stability and operation stability of the device are also significantly improved. (The efficiency remains at 91% of the original for 800 h of atmospheric storage; the efficiency remains at 92% of the original for 200 h of continuous light exposure.) The present strategy realizes the simultaneous improvement of photovoltaic properties and stability of CsPbI_3_ QD solar cells and also provides a reference for surface ligand engineering to realize highly efficient and stable perovskite quantum dot solar cells.

## 1. Introduction

The inorganic halide perovskite material CsPbX_3_ (X = I, Br, Cl) has excellent photovoltaic properties and thermal stability, among which the most suitable for photovoltaic applications is CsPbI_3_ with a suitable band gap [1,2]. However, the cubic phase CsPbI_3_ perovskite films are prone to phase transitions to non−photovoltaic phases at room temperature [3,4,5]. Kovalenko et al. prepared CsPbI_3_ perovskite materials with nanoscale dimensions, and by increasing the surface energy of the crystalline structure, they achieved higher cubic phase stability for CsPbI_3_ quantum dots (or nanocrystals) than for native films [6]. Meanwhile, the non−radiative composite channel was significantly suppressed compared to the bulk thin film, and the photoluminescence yield of the quantum dot material was close to unity [7,8]. Due to the advances in solar cell materials and device design, the power conversion efficiency of CsPbI_3_ perovskite quantum dot solar cells (PQSCs) has been increased from 10.77% to 16.2% in a short period of time, and they exhibit excellent open−circuit voltage characteristics [9,10,11,12,13].

In spite of the excellent photovoltaic properties and phase stability of CsPbI_3_ quantum dots, the efficiency of current solar devices is still relatively low, with a large gap compared to devices based on thin−film technology. To stabilize the CsPbI_3_ cubic phase and prevent agglomeration during the preparation process, current CsPbI_3_ quantum dots synthesized using thermal injection usually introduce oleic acid with long−chain properties as an organic shell layer through surface passivation and modification [14]. However, the insulating properties of the long−chain ligands inhibit charge exchange between neighboring QDs and affect the electrical properties after filming [15]. In the device fabrication process, the common method is to prepare quantum dot films using layer−by−layer deposition, and each layer is cleaned with a washing solvent (methyl acetate (MeOAc)) after deposition to ensure the removal of the long−chain ligands [9]. However, given the low polarity (dielectric constant) of MeOAc, sufficient OA removal cannot be achieved during cleaning [16]. As a result, the corresponding solar cell devices exhibit low device efficiencies because the overall electrical transport properties of the QD films are still limited.

Various types of ligand modification strategies have been proposed by researchers to improve the film transport properties, mainly through the introduction of coordinative organic groups and short−chain molecules in the washing solvent to realize the re−coordination of the quantum dot surface, for example, the introduction of formamidine iodide (FAI) dissolved in ethyl acetate (EtOAc) to prepare saturated solutions for PQD film post−treatment [10]; introduction of, e.g., triphenyl phosphite and phenyl ammonium as a replacement for OA [17,18]; introduction of multifunctional ligands glycosamine (GLA) and 4−aminobenzoic acid (4−ABA) to achieve quantum dot surface passivation and act as lattice anchors [19]. 2−Pentanol (2−PeOH) with suitable dielectric constant and acidity was introduced to completely remove long−chain OA [20]. However, considering the lower solubility of the low−polarity ethyl acetate (EtOAc) for the short−chain ligands, the effect of achieving long−chain replacement during the cleaning process of quantum dot thin film deposition on a layer−by−layer basis is still unsatisfactory [10]. More suitable strategies are necessary for the achievement of high−quality film preparation.

In order to address this problem, a ligand modification strategy for CsPbI_3_ quantum dots based on the benzylphosphonic acid (BPA) medium is proposed in this project, which is a two−step “preparation−film formation” strategy. First, during the preparation process, short−chain BPA with a P=O group was introduced into the quantum dots crude solution to realize the initial passivation and long−chain substitution on the surface. Then, during the layer−by−layer preparation of QD films, the secondary surface modification was achieved by adding BPA into the washing solvent to completely remove the long chains and protect the interface with passivation, which effectively passivated the surface defects of the QDs, inhibited the non−radiative complexation, and further ensured their high phase stability properties. In addition, the short−chain ligands can promote charge exchange between neighboring quantum dots inside the perovskite film, which further improves the photoelectric properties of the film. Finally, the BPA−modified perovskite solar cell achieved a significant efficiency improvement, with the optimal device achieving 13.91% power conversion efficiency (11.41% for the reference cell). In addition, device stability was also significantly improved. The device can retain 91% of its original efficiency after 800 h of storage in the atmosphere and 92% of its original efficiency after 200 h of continuous light irradiation in the device. This stepwise−process−controlled ligand modification strategy achieves a simultaneous improvement in the photoelectric properties and stability of CsPbI_3_ perovskite quantum dots and also shows the way for surface ligand engineering to realize highly efficient and stable quantum dot perovskite solar cells.

## 2. Experimental Details

### 2.1. Materials

*N*,*N*′−dimethylformamide (DMF), dimethyl sulfoxide (DMSO), IPA, and SnO_2_ colloidal precursors were purchased from Alfa Aesar. Lead Iodide (PbI_2_, 99.999%), Carbonate Cesium (Cs_2_CO_3_, 99.99%), benzyl phosphonic acid, methyl acetate anhydrate (99.99%), toluene anhydrate, octane anhydrate, oleic acid (80~90), oleylamine (70%), spiro−OMeTAD, lithium bis(trifluoromethanesulfonyl)imide (Li−TFSI), tertiary−butylpyridine (tBP) were purchased from Sigma−Aldrich (Shanghai, China) and from Alfa Aesar. Oleic acid and oleylamine were degassed at 100 °C for 1 h before use.

### 2.2. Synthesis and Post−Treatment of CsPbI_3_ Quantum Dots

The original CsPbI_3_ QD was synthesized according to the reported hot injection method with minor modifications [21]. Firstly, Cs−Oleat was prepared by loading Cs_2_CO_3_ (0.814 g), oleic acid (2.5 mL), and ODE (40 mL) into a three−necked flask and degassing at 120 °C for 1 h. After that, the reaction temperature was increased to 150 °C under N_2_ flow until the solid was totally dissolved, then cooled down, and the clean solution was kept in a N_2_ atmosphere.

PbI_2_ (0.552 g) and ODE (30 mL) were loaded into a 50 mL 3−necked flask and dried under vacuum for 1 h at 120 °C. Dried oleylamine (3 mL) and oleic acid (3 mL) were injected into the flask under N_2_ flow. After the PbI_2_ was completely soluble, the temperature was raised to 165 °C, and 2.4 mL of preprepared oleate−Cs was quickly injected; 7 s later, the reaction was cooled with an ice−water bath. The crude PQD solution was kept for the following cleaning and post−treatment procedures.

CsPbI_3_ PQD post−treatment:

An amount of 6 mL of the crude PQD was transferred to a 50 mL centrifugate tube; then, 12 mL methyl acetate (with or without benzylphosphonic acid, BPA) was added, followed by centrifugation at 8500 rpm for 5 min, and the precipitates were collected and redispersed in 2 mL toluene. The second cycle cleaning was conducted by adding 3 mL methyl acetate into 2 mL toluene and centrifugating again at 8500 rpm for 5 min, and the precipitate was redispersed in octane for storage.

Preparation of CsPbI_3_ quantum dot active layer:

Here, perovskite quantum dot light−absorbing layers (active layer) were prepared using a layer−by−layer deposition technique. Each layer of CsPbI_3_ PQD was spin−coated with PQD solution (85 mg/mL in octane) at 1000 rpm for 10 s and 2000 rpm for 25 s. MeOAc was used as a washing solvent which was added dropwise to the film for 3 s and then spin−coated at 2000 rpm for 30 s followed by drying. The procedure above was repeated four times to obtain films 400 nm in thickness. Finally, pure MeOAc and MeOAc incorporated with benzylphosphonic acid were added to the initial PQD films and left to rest for 5 s, rotated at 1000 rpm for 10 s, and rotated at 2000 rpm for 30 s, followed by drying. Both BPA−modified and unmodified films were obtained.

### 2.3. Device Fabrication

The ITO substrate was sequentially cleaned with deionized water, acetone, ethanol, and isopropanol. SnO_2_ was dropped onto the substrate for spin−coating (2500 rpm, 40 s) and annealed at 155 °C for 25 min in ambient air. The ITO/ SnO_2_ substrate was treated with UVO for 10 min after cooling to room temperature. Then, the PQD active layer was deposited according to the prementioned procedure. A total of 100 mg of Spiro−OMeTAD, 28.5 μL of tBP, 17.8 μL of Li−TFSI/ACN (520 mg/mL dissolved in ACN), 20 μL of FK209/ACN (200 mg/mL dissolved in ACN) were added to 1 mL of CB on top of the PQD film and stirred for 4 h to prepare the Spiro−OMeTAD precursor solution. This Spiro−OMeTAD precursor solution was coated at 3200 rpm for 30 s on the perovskite layer. At last, 3 nm MoO_3_ and 60 nm Ag were deposited using thermal evaporation with an area of 0.0706 cm^2^ for each device. The fabrication of the films and devices was done in a glove box (25% relative humidity atmosphere).

### 2.4. Characterization

The absorption and PL spectra were obtained with a UV−Vis spectrophotometer (HITACHI U−3010, Kobe, Japan) and a Fluorescence Spectrometer (Fluoromax−4 spectrofluometer), respectively. A transmission electron microscope (JEM−1400F TEM) was used to investigate the morphology and crystallinity of the quantum dots. Assessment of the crystalline structure on the ITO substrate was performed using an X−ray diffractometer (D/MAX−2400, Rigaku, Japan) with Cu Kα radiation. The surface anchoring of ligand species on QD was recorded using Fourier transform infrared spectroscopy (FTIR, Nicolet iS50). The chemical bonding state of the perovskite films was studied using XPS (Thermo Fisher, ESCALAB Xi+, Waltham, MA, USA) with an Al Kg X−ray source. The XPS spectra were calibrated using the binding energy of C 1 s. The photovoltaic performance was estimated under a AAA solar simulator (Newport, 94063A, Cheshire, CW1 6AG, UK), with AM 1.5G irradiation with an intensity of 100 mW cm^−2^. The photocurrent–voltage (J−V) curve was measured by a Keithley (2400 Series Sourcemeter), and the scan rates were 0.15 V s^−1^ starting from 1.3 V to −0.1 V. Here, the J−V curve of the device is measured in air. Incident photon−to−current conversion efficiency (IPCE) spectra were collected at the AC mode using the solar cell quantum efficiency measurement system (SolarCellScan 100, Zolix instruments. Co., Ltd., Beijing, China).

## 3. Results and Discussion

### 3.1. Characterization of CsPbI_3_ QDs

**Figure 1:**
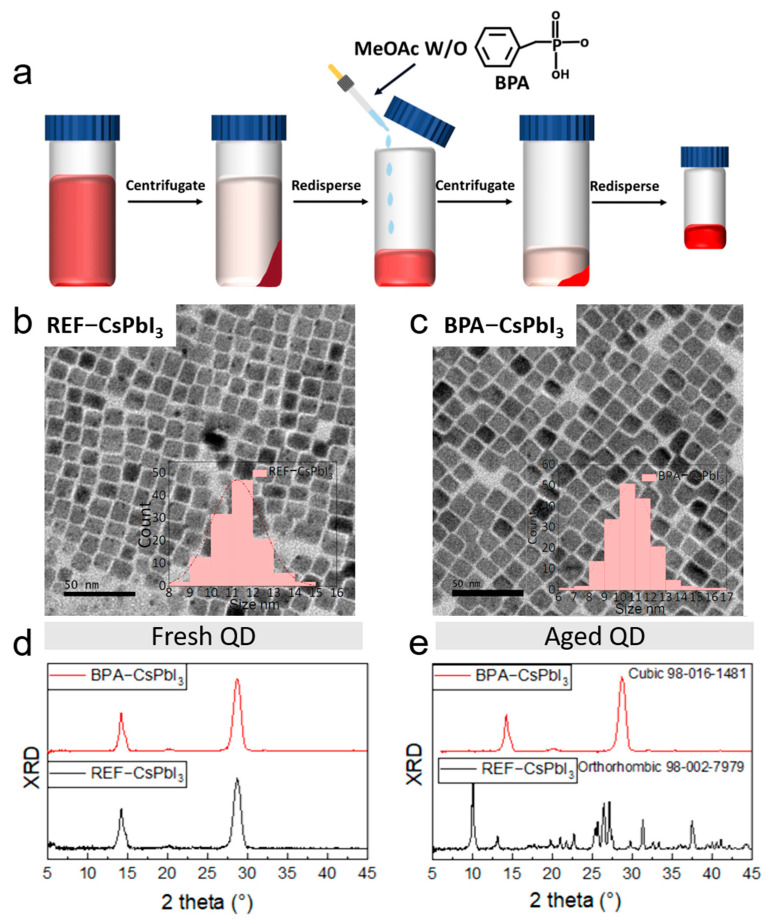
(**a**) Synthesis and modification process of CsPbI_3_ QDs. (**b**,**c**) Transmission electron microscopy (TEM) images and statistical distribution of diameters of two kinds of QDs. XRD results of fresh (**d**) and aged (**e**) QDs.

The reference colloidal CsPbI_3_ perovskite QDs sample, which was capped with OA^−^ and OAM^+^ (named as REF−CsPbI_3_ Figure S1) in this work, was synthesized using the conventional hot injection method with some modification and purified with sample methyl acerate (MeOAc) to remove the excess ligands and residuals of the octadecene solvent (see experimental section). The post−treated BPA sample BPA−CsPbI_3_ was obtained by introducing the BPA−MeOAc antisolvent into the crude QD solution during the second cycle of the QD purifying procedure (as described in Figure 1a). In order to investigate the effect pattern of BPA introduction on the material properties and optical properties of −CsPbI_3_ QDs, the amount of BPA added to the MeOAc was varied from 0 mg/mL to 7 mg/mL. Figure S1 shows the ultraviolet–visible absorption and the photoluminescence (PL) spectroscopy of BPA−CsPbI3 samples treated with different dosages of BPA. The absorbance edge of the UV−vis spectra of the REF−CsPbI_3_ colloidal solution set on at 690 nm and the PL emission peak was centered at 680 nm. The absorption and PL peaks of the BPA−treated samples were slightly blueshifted when compared with reference one (Figure S2). The shift of the PL emission is due to the improved electronic coupling properties between the PQDs after the partial replacement of the long−chain OA ligand by a short−chain BAP ligand [22].

The other obvious phenomenon is that the PL emission intensity of the CsPbI_3_ colloidal solution displays an increasing trend and then decreases as the dosage of BPA increases (Figure S3). This can be attributed to the fact that coordinating BPA can affect both the surface defects of PQDs and increase the electronic coupling between interacting PQDs, causing changes in PL intensity [23,24]. To further confirm the optimal dose of BPA, the photoluminescence quantum yield of (PLQY) was measured. The result indicated that the PLQY of the fresh QD solution was 76%, 85%, 97%, 90%, and 83%. The photoluminescence property of the reference CsPbI_3_ without BPA treatment dropped sharply after 5 days of aging (Figure S4). Otherwise, the BPA−treated sample maintained its PLQY; especially for the sample treated with the 3 mg/mL BPA MeOAc solution, the PLQY remained at 95% of its initial value. Even after 1 month, the PLQY remained at 80% of the baseline. After evaluating the PLQY of fresh and aged colloidal samples varying in BPA amount, the sample treated with 3 mg/mL MeOAc was assigned as the priority one. Thereinafter, we named BPA−CsPbI_3_ the best−performing BPA−treated sample and conducted a comprehensive comparison between it and the reference sample (REF−CsPbI_3_).

The QD morphology and phase structure were studied with transmission electron microscopy (TEM) and X−ray diffraction patterns. The morphology of the REF−CsPbI_3_ and BPA−CsPbI_3_ QDs can be seen in Figure 1b,c; both QDs are monodisperse and uniform in size with little variety. The average sizes of the BPA−CsPbI_3_ QDs and REF−CsPbI_3_ are 10.9 nm and 11.5 nm, respectively. Similarly, there were almost no differences between the XRD patterns (Figure 1d,e) of the reference and the BPA−modified fresh sample. The diffraction peaks of the QD sample all correspond to the cubic phase of the CsPbI_3_ crystal [6]. However, as time varied, the diffraction peak related to the orthorhombic phase of CsPbI_3_ present in the sample of REF−CsPbI_3_, while the crystal structure of PBA−CsPbI_3_ remained in the cubic phase [25], which suggests the BPA passivated the surface of CsPbI_3_ and enhanced the phase stability of CsPbI_3_ QDs. The high PLQY of aged BPA−CsPbI_3_ could also have contributed to its advantage in stability.

**Figure 2:**
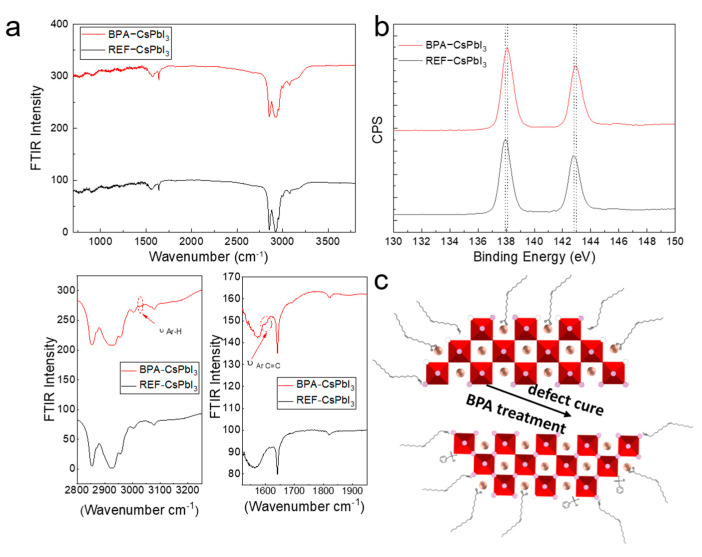
(**a**) FTIR and (**b**) XPS results of reference and BPA−modified CsPbI_3_ QDs. (**c**) Schematic diagram of BPA ligands modifying CsPbI_3_ QDs.

The passivation of BPA to the CsPbI_3_ QD surface is further confirmed by the Fourier transform infrared spectroscopy (FTIR), where the distinct C=C stretching vibration owing to the benzene ring was centered at 1588 cm^−1^ and the C−H vibration at 3027 cm^−1^ attributed to the aromatic ring appears in the FTIR curve of the BPA−CsPbI_3_ film (Figure 2a), indicating that the BPA is likely anchored to BPA−CsPbI_3_ QD surfaces. X−ray photoelectron spectroscopy (XPS) was also performed to study the surface composition of PQDs, and the result is shown in Figure 2b. The increased binding energy of Pb 4f in the BPA−treated sample compared to the reference one demonstrates more coordinated lead species due to P=O group contributions from the BPA [26,27] (Figure 2c).

**Figure 3:**
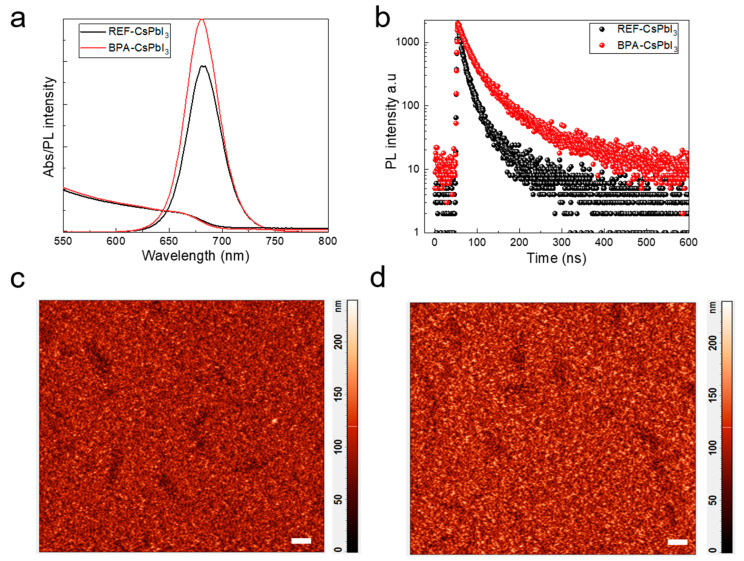
(**a**) Representative steady−state PL and (**b**) TRPL decay curves of two CsPbI_3_ QDs films deposited on glass. (**c**,**d**) AFM topography images of two CsPbI_3_ QDs films on ITO/SnO_2_ substrate; scale bar is 2 µm.

Afterwards, we investigated the BAP treatment’s effect on the properties of CsPbI_3_ QD film. The optical property study of the QD film samples was performed using UV−vis and PL spectroscopy. As shown in Figure 3a, the absorption edges of these PQD films have no obvious differences, and the PL emission peak center is located at 680 nm. The small stock shift illustrates the good quality and uniformity of the synthesized QDs and film. Based on the absorption and photoluminescence results, the band gap of the QDs can be determined to be 1.8 eV. It can be seen that the PL intensity of the BPA−treated PQDs is increased compared to the pure PQDs. This suggests that the BPA ligand can effectively inhibit the surface defects of non−radiative recombination by passivating the surface defects of PQDs, thus effectively inhibiting non−radiative recombination. Meanwhile, it can effectively fill the vacancies on the surface of PQDs, reduce the erosion of moisture and oxygen, and improve the stability of PQD films.

Meanwhile, time−resolved photoluminescence (TRPL) spectroscopy was applied to investigate the photoexcited carrier dynamics of the CsPbI_3_ QDs films. Time−resolved photoluminescence (TRPL) spectroscopy (the fitting process is described in the Supplementary Information) was carried out (see Figure 3b). τ_1_ and τ_2_ of the perovskite films are summarized in Table S1. Here, τ_1_ is the fast decay, relating to the recombination of the traps assisting, and τ_2_ is the slow decay, relating to the recombination of the inside of the perovskite film. The average decay lifetime (fitted with double exponential decay) extends from 30.42 ns for REF−CsPbI_3_ to 50.11 ns for PBA−CsPbI_3_ (Figure 3b), suggesting that the surface of the BPA−CsPbI_3_ QDs is better passivated than that of the REF−CsPbI_3_ samples, which is in agreement with the aforementioned properties such as PLQY and phase stability.

Film morphology also influences the electrical transport in solar cell devices. Here, the atomic force microscopy (AFM) technique was used for the morphological study of QD films, and the relevant results are shown in Figure 3c,d. It can be seen that both REF and BPA samples show reasonable homogeneity. The surface of the BPA−modified film was smooth, with a root mean square (RMS) of 16.32 nm, compared to the roughness (RMS = 22.14) of the REF film, which can be attributed to the fact that the short−chain ligand effectively replaces the naturally occurring long−chain OA, which facilitates the tight alignment of the QDs and thus is more favorable for charge transfer between the device interfaces.

### 3.2. Device Performance and Stability Analysis

**Figure 4:**
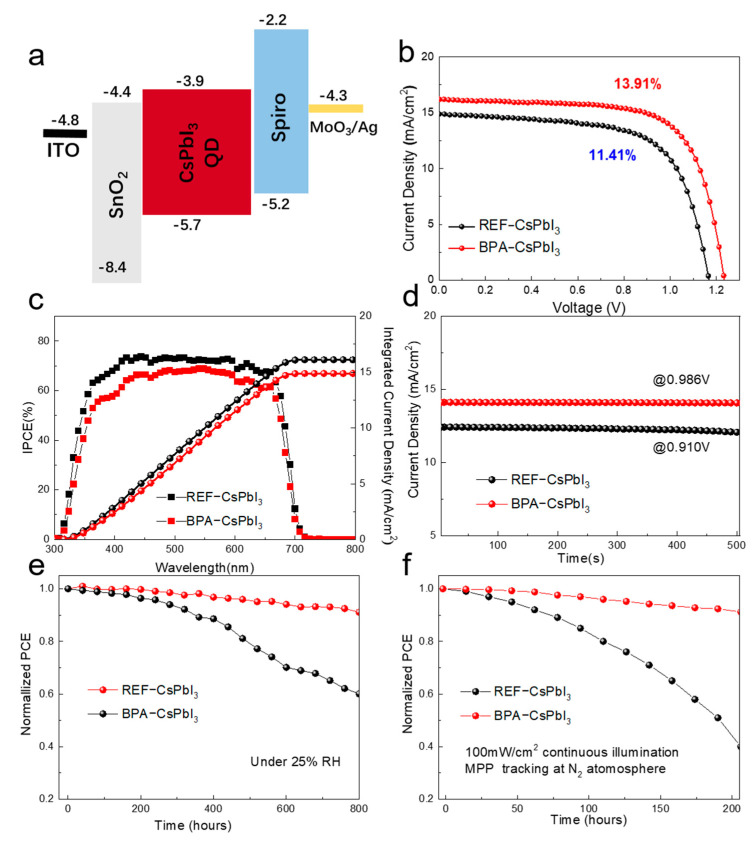
(**a**) Device structure diagram of PQSCs. (**b**) Representative J−V curves of PQSCs. (**c**) Stabilized current density output of pristine and BPA−modified PQSCs measured at MPP voltage. (**d**) EQE spectra and integrated J_SC_ for pristine and BPA−modified PQSCs. (**e**) Storage stability and (**f**) light−soaking stability of pristine and BPA−modified PQSCs.

Based on the analysis of the physical properties of perovskite materials and films, it can be seen that introducing phenyl can effectively passivate the surface defects of CsPbI_3_ quantum dots and improve their stability. In order to investigate the effect of the positively acting phenyl on the performance of photovoltaic devices, planar−structured perovskite quantum dot solar cell devices (PQSCs) of ITO/SnO_2_/CsPbI_3_ QD/spiro−OMeTAD/MoO_3_/Ag were designed in this work. The structure is shown in Figure 4a. The light−absorbing film was prepared using layer−by−layer deposition, and the specific process used to prepare the device can be found in the supporting information. The J−V curves of the control and BPA−modified PSCs were first measured under standard AM 1.5 G illumination (100 mW/cm^2^), and the photovoltaic performance parameters of the champion are shown in Figure 4b. The power conversion efficiency (PCE) of the control device is only 11.41%. The open circuit voltage (V_OC_) is 1.17 V, the short circuit current density (J_SC_) is 14.98 mA/cm^2^, and the fill factor (FF) is 64.92%. In contrast, the device parameters are significantly higher for the devices with the BPA modification strategy. The champion device had a PCE of 13.91%, a J_SC_ of 16.18 mA/cm^2^, a V_OC_ of 1.24 V, and a FF of 69.48%. This is also a competitive efficiency achievement in the current work on CsPbI_3_ PQSCs (summarized in Table S2). Here, the introduction of short−chain ligands, which can effectively passivate the surface defects of CsPbI_3_ quantum dots and thus inhibit the non−radiative complexation, is mainly responsible for the efficiency enhancement of the BPA−modified device, as manifested by the increase in V_OC_ and FF. The statistical values of PCE are shown in Figure S5 and Table S3, where 30 individual samples were fabricated for each device structure to evaluate their reproducibility. These devices are highly reproducible and have a narrow distribution of BPA−modified PCE, as shown by the statistical results. Figure 4c shows the incident photoelectron conversion efficiency (IPCE) spectra of the control and BPA−modified devices. The integrated J_SC_s are 17.54 mA/cm^2^ and 21.98 mA/cm^2^, respectively, which are in good agreement with the short circuit current density (J_SC_) obtained from the J−V measurements. Figure 4d shows the steady−state photocurrents at the maximum power point of 500 s for the control and the BPA−modified devices, with the latter showing a more stable power output.

The stability of photovoltaic devices is also an important factor in making them viable to apply and industrialize. Here, we also investigate their placement. Figure 4e shows the stability of the two types of devices under atmospheric atmosphere (25% RH) conditions without encapsulation. The BPA−modified device retained 91% of its initial efficiency after 800 h of storage. The control device retained only 60% of its initial efficiency after the same period. Figure 4f shows the operational stability of the two types of devices in a room−temperature N_2_ atmosphere (100 mW/cm^2^ continuous light under MPP tracking) without encapsulation. The BPA−modified devices maintained 92% of the initial efficiency for 200 h of continuous operation, while the control devices showed a rapid loss of PEC within 100 h. The BPA−modified devices were also found to be stable at room temperature in the N_2_ atmosphere for the same period of time. Therefore, this satisfactory long−term operation in BPA−modified PQSCs is attributed to the improved properties of perovskite QDs, such as effective surface defect healing and surface wrapping. These advantages suppress the phase transition from the perovskite phase to the non−perovskite phase, while effectively minimizing the penetration of moisture and oxygen into the perovskite material. As a result, the degradation of device performance is mitigated.

### 3.3. Mechanism Analysis of Defect Passivation and Charge Transport

The study of the performance of the device shows that the introduction of BPA can increase the V_OC_, FF, and J_SC_ of the device simultaneously. The increase in V_OC_ and FF can be attributed to the fact that the BPA ligands can effectively coordinate with and passivate the Pb on the surface of CsPBI_3_ quantum dots, which reduces the non−radiative recombination [28,29]. While the enhancement of the J_SC_ is affected by the charge complexation behavior and charge transport behavior in the device, further analysis of the reasons for the performance improvement is necessary.

**Figure 5:**
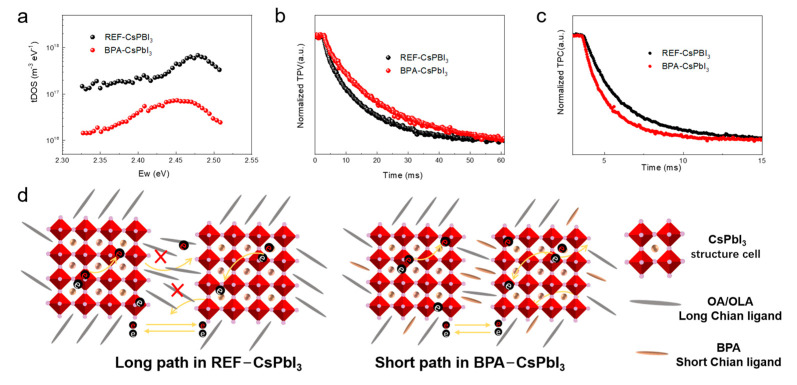
(**a**) Trap density of states (tDOS) of the devices with the reference/modified perovskite QDs; (**b**) TPV and (**c**) TPC measurement of pristine and BPA−modified PQSCs; (**d**) the mechanism diagram of charge exchange in different perovskite QD films.

In order to investigate the reason for the V_OC_/FF enhancement of modified perovskite quantum dot devices, we measured the defect density of states (tDOS) distribution of pristine and modified PQSCs using thermal conductivity spectroscopy (TAS) [30]. TAS is a well−established and effective analytical method to derive the trap density of states in the device with impedance spectroscopy (see Supplementary Information) [31,32]. The tDOS of PSCs based on BPA ligand−modified membranes decreased from ~10^17^ m^−3^ eV^−1^ to ~10^16^ m^−3^ eV^−1^, almost an order of magnitude compared to the reference device, as shown in Figure 5a. The decrease in tDOS is consistent with the limitation of non−radiative charge complexation. This can be attributed to the effective passivation of the short−chain ligands on the surface of CsPBI_3_ quantum dots.

In response to the fact that the J_SC_ performance of the BPA−assisted device is superior to that of the unmodified standard device, the altered charge complexation and charge transport properties inside the device need to be further analyzed. Here, transient photocurrent (TPC) photovoltage (TPV) and transient photocurrent (TPC) techniques are employed to analyze the charge behavior inside the device, and the results are shown in Figure 5b,c, respectively [33]. The BPA−−modified PQSC exhibits a longer TPV lifetime than that of the control PQSC, which is a reflection of the effective suppression of the non−radiative recombination. Here as well, the TPV results are in good agreement with the XPS and t−DOS analyses, elucidating the defect passivation effect of the ligand modification. This is reflected in the improvement of the Voc and FF in the device. For the TPC measurements, the BPA−modified device showed a TPC lifetime (1.68 µs) significantly lower than that of the control device (2.03 µs) (shown in Figure 5c). It is well known that the TPC lifetime corresponds to the charge transport efficiency in the device. Thus, the BPA−modified PSCs had a more efficient charge transport process in comparison to the control PSCs.

Overall, the modification of quantum dots with BPA ligands is reflected in the following two aspects: For pristine CsPbI_3_ QD films that are only modified with long−chain OA, there is strong non−radiative recombination and inefficient charge transport. This is because the long−chain insulating OA cannot provide a transport channel for the charge to exchange between neighboring QDs. In contrast, a fast charge transport channel effect can be realized with the short−ligand BPA. (See Figure 5d for the schematic diagram.) Therefore, the short−chain and highly efficient coordination ability of BPA has the dual advantages of defect passivation and charge transport compared to the widely used long−chain OA ligands.

## 4. Conclusions

The long−chain insulating ligands introduced during the preparation of CsPbI_3_ chalcogenide quantum dots lead to low electrical transport properties of quantum dot films, which affects the efficiency of solar cells. To address the above issue, a dual ligand handling strategy is employed in this work during the quantum dot preparation process and the quantum dot film formation process. The defect state density and inter−cell transport properties on the quantum dot surface are improved by using short−chain Benzenephosphonic acid. Repassivation of the QDs can effectively inhibit non−radiative recombination, while the short−chain ligands can promote efficient charge exchange between neighboring QDs. Based on this “dual−process” ligand management strategy, a PQSC efficiency of 13.91% is finally achieved. The storage stability and operational stability of the device are also significantly improved.

## Data Availability

Data is contained within the article.

## Supplementary Materials

The following supporting information can be downloaded at: https://www.mdpi.com/article/10.3390/nano13233032/s1, Figure S1: Photograph of as Synthesized CspbI_3_ nanocrystals; Figure S2: PL and UV-Vis absorption spectra of CsPbI3 treated with different dose of BPA; Figure S3: PL intensity of CsPbI_3_ sample treated with different dose of BPA at the same optical density; Figure S4: Photograph of CsPbI_3_ sample treated with different dose of BPA and is varying with aging time; Figure S5: The statistical Photovoltaic Parameters of pristine and BPA-modified PQSCs. Table S1: Parameters of the time-resolved photoluminescence (TRPL) spectroscopy of control and BPA-modified perovskite films; Table S2: Device design strategies and performance of CsPbl3 PQSCs work recently. Table S3: The statistical Photovoltaic Parameters of pristine and BPA-modified PQSCs. References [8,20,34,35,36,37] are cited in the supplementary materials.

## References

[1] National Renewable Energy Laboratory Best Research−Cell Efficiency Chart. https://www.nrel.gov/pv/cell−efficiency.html.

[2] Park J., Kim J., Yun H.S., Paik M.J., Noh E., Mun H.J., Kim M.G., Shin T.J., Seok S.I. (2023). Controlled growth of perovskite layers with volatile alkylammonium chlorides. Nature.

[3] Eperon G.E., Paternò G.M., Sutton R.J., Zampetti A., Haghighirad A.A., Cacialli F., Snaith H.J. (2015). Inorganic caesium lead iodide perovskite solar cells. J. Mater. Chem. A.

[4] Stoumpos C.C., Frazer L., Clark D.J., Kim Y.S., Rhim S.H., Freeman A.J., Ketterson J.B., Jang J.I., Kanatzidis M.G. (2015). Hybrid Germanium Iodide Perovskite Semiconductors: Active Lone Pairs, Structural Distortions, Direct and Indirect Energy Gaps, and Strong Nonlinear Optical Properties. J. Am. Chem. Soc..

[5] Mubiayi K.P., Moloto N., Moloto M.J. (2018). Effect of diphenylphosphinic acid on cesium lead iodide perovskite stability. Crystengcomm.

[6] Protesescu L., Yakunin S., Bodnarchuk M.I., Krieg F., Caputo R., Hendon C.H., Yang R.X., Walsh A., Kovalenko M.V. (2015). Nanocrystals of Cesium Lead Halide Perovskites (CsPbX, X = Cl, Br, and I): Novel Optoelectronic Materials Showing Bright Emission with Wide Color Gamut. Nano Lett..

[7] Mei X.Y., Jia D.L., Chen J.X., Zheng S.Y., Zhang X.L. (2022). Approaching high−performance light−emitting devices upon perovskite quantum dots: Advances and prospects. Nano Today.

[8] Jia D.L., Chen J.X., Yu M., Liu J.H., Johansson E.M.J., Hagfeldt A., Zhang X.L. (2020). Dual Passivation of CsPbI Perovskite Nanocrystals with Amino Acid Ligands for Efficient Quantum Dot Solar Cells. Small.

[9] Swarnkar A., Marshall A.R., Sanehira E.M., Chernomordik B.D., Moore D.T., Christians J.A., Chakrabarti T., Luther J.M. (2016). Quantum dot−induced phase stabilization of α−CsPbI perovskite for high−efficiency photovoltaics. Science.

[10] Sanehira E.M., Marshall A.R., Christians J.A., Harvey S.P., Ciesielski P.N., Wheeler L.M., Schulz P., Lin L.Y., Beard M.C., Luther J.M. (2017). Enhanced mobility CsPbI quantum dot arrays for record−efficiency, high−voltage photovoltaic cells. Sci. Adv..

[11] Wang Y., Yuan J.Y., Zhang X.L., Ling X.F., Larson B.W., Zhao Q., Yang Y.G., Shi Y., Luther J.M., Ma W.L. (2020). Surface Ligand Management Aided by a Secondary Amine Enables Increased Synthesis Yield of CsPbI Perovskite Quantum Dots and High Photovoltaic Performance. Adv. Mater..

[12] Yuan J.B., Zhang X.L., Sun J.G., Patterson R., Yao H.F., Xue D., Wang Y., Ji K., Hu L., Huang S.J. (2021). Hybrid Perovskite Quantum Dot/Non−Fullerene Molecule Solar Cells with Efficiency Over 15%. Adv. Funct. Mater..

[13] Shi J.W., Li F.C., Jin Y., Liu C., Cohen−Kleinstein B., Yuan S., Li Y.Y., Wang Z.K., Yuan J.Y., Ma W.L. (2020). In Situ Ligand Bonding Management of CsPbI Perovskite Quantum Dots Enables High−Performance Photovoltaics and Red Light−Emitting Diodes. Angew. Chem. Int. Edit.

[14] Zhang Y.N., Siegler T.D., Thomas C.J., Abney M.K., Shah T., De Gorostiza A., Greene R.M., Korgel B.A. (2020). A “Tips and Tricks” Practical Guide to the Synthesis of Metal Halide Perovskite Nanocrystals. Chem. Mater..

[15] Ling X.F., Yuan J.Y., Zhang X.L., Qian Y.L., Zakeeruddin S.M., Larson B.W., Zhao Q., Shi J.W., Yang J.C., Ji K. (2020). Guanidinium−Assisted Surface Matrix Engineering for Highly Efficient Perovskite Quantum Dot Photovoltaics. Adv. Mater..

[16] Wheeler L.M., Sanehira E.M., Marshall A.R., Schulz P., Suri M., Anderson N.C., Christians J.A., Nordlund D., Sokaras D., Kroll T. (2018). Targeted Ligand−Exchange Chemistry on Cesium Lead Halide Perovskite Quantum Dots for High−Efficiency Photovoltaics. J. Am. Chem. Soc..

[17] Kim J., Cho S., Dinic F., Choi J., Choi C., Jeong S.M., Lee J.S., Voznyy O., Ko M.J., Kim Y. (2020). Hydrophobic stabilizer−anchored fully inorganic perovskite quantum dots enhance moisture resistance and photovoltaic performance. Nano Energy.

[18] Zhang X.L., Huang H.H., Maung Y.M., Yuan J.Y., Ma W.L. (2021). Aromatic amine−assisted pseudo−solution−phase ligand exchange in CsPbI perovskite quantum dot solar cells. Chem. Commun..

[19] Ding S.S., Steele J.A., Chen P., Lin T.E., He D.X., Zhang C.X., Fan X.Q., Solano E., Whittaker A.K., Hao M.M. (2023). Ligand−Mediated Homojunction Structure for High−Efficiency FAPbI Quantum Dot Solar Cells. Adv. Energy Mater..

[20] Jia D.L., Chen J.X., Qiu J.M., Ma H.L., Yu M., Liu J.H., Zhang X.L. (2022). Tailoring solvent−mediated ligand exchange for CsPbI perovskite quantum dot solar cells with efficiency exceeding 16.5%. Joule.

[21] Jia D.L., Chen J.X., Mei X.Y., Fan W.T., Luo S., Yu M., Liu J.H., Zhang X.L. (2021). Surface matrix curing of inorganic CsPbI perovskite quantum dots for solar cells with efficiency over 16%. Energy Environ. Sci..

[22] Koole R., Liljeroth P., Donegá C.D., Vanmaekelbergh D., Meijerink A. (2006). Electronic coupling and exciton energy transfer in CdTe quantum−dot molecules. J. Am. Chem. Soc..

[23] Khan J., Zhang X.L., Yuan J.Y., Wang Y., Shi G.Z., Patterson R., Shi J.W., Ling X.F., Hu L., Wu T. (2020). Tuning the Surface−Passivating Ligand Anchoring Position Enables Phase Robustness in CsPbI Perovskite Quantum Dot Solar Cells. ACS Energy Lett..

[24] Vickers E.T., Graham T.A., Chowdhury A.H., Bahrami B., Dreskin B.W., Lindley S., Naghadeh S.B., Qiao Q.Q., Zhang J.Z. (2018). Improving Charge Carrier Delocalization in Perovskite Quantum Dots by Surface Passivation with Conductive Aromatic Ligands. ACS Energy Lett..

[25] Yao J.S., Ge J., Wang K.H., Zhang G., Zhu B.S., Chen C., Zhang Q., Luo Y., Shu H., Yao H.B. (2019). Few−nanometer−sized α−CspbI_3_ quantum dots enabled by strontium substitution and iodide passivation for efficient red−light emitting diodes. J. Am. Chem. Soc..

[26] Zhang J.B., Yin C.Y., Yang F., Yao Y., Yuan F.L., Chen H.T., Wang R.W., Bai S., Tu G.L., Hou L.T. (2021). Highly Luminescent and Stable CsPbI Perovskite Nanocrystals with Sodium Dodecyl Sulfate Ligand Passivation for Red−Light−Emitting Diodes. J. Phys. Chem. Lett..

[27] Shen W., Dai Y.J., Cai B., Chen S., Yang H., Ma Y.Z., Chen Y.F., Su Z., Zhang J.B., Qiu Y. (2023). Ligand−Assisted Breaking Crystal Symmetry to Achieve Stable γ−CsPbI Nanorods with Strong Polarization Response. ACS Energy Lett..

[28] Luo D.Y., Su R., Zhang W., Gong Q.H., Zhu R. (2020). Minimizing non−radiative recombination losses in perovskite solar cells. Nat. Rev. Mater..

[29] Li B., Chang B.H., Pan L., Li Z.H., Fu L., He Z.B., Yin L.W. (2020). Tin−Based Defects and Passivation Strategies in Tin−Related Perovskite Solar Cells. ACS Energy Lett..

[30] Zhang F., Bi D.Q., Pellet N., Xiao C.X., Li Z., Berry J.J., Zakeeruddin S.M., Zhu K., Grätzel M. (2018). Suppressing defects through the synergistic effect of a Lewis base and a Lewis acid for highly efficient and stable perovskite solar cells. Energy Environ. Sci..

[31] Shao Y.H., Xiao Z.G., Bi C., Yuan Y.B., Huang J.S. (2014). Origin and elimination of photocurrent hysteresis by fullerene passivation in CHNHPbI_3_ planar heterojunction solar cells. Nat. Commun..

[32] Bi C., Wang Q., Shao Y.C., Yuan Y.B., Xiao Z.G., Huang J.S. (2015). Non−wetting surface−driven high−aspect−ratio crystalline grain growth for efficient hybrid perovskite solar cells. Nat. Commun..

[33] Sun J.G., Li B., Hu L., Guo J.J., Ling X.F., Zhang X.L., Zhang C., Wu X.X., Huang H.H., Han C.X. (2023). Hybrid Block Copolymer/Perovskite Heterointerfaces for Efficient Solar Cells. Adv. Mater..

[34] Yuan J., Ling X., Yang D., Li F., Zhou S., Shi J., Qian Y., Hu J., Sun Y., Yang Y. (2018). Band−Aligned Polymeric Hole Transport Materials for Extremely Low Energy Loss α−CsPbI_3_ Perovskite Nanocrystal Solar Cells. Joule.

[35] Liu Y., Zhao X., Yang Z., Li Q., Wei W., Hu B., Chen W. (2020). Cu_12_Sb_4_S_13_ Quantum Dots with Ligand Exchange as Hole Transport Materials in All−Inorganic Perovskite CsPbI_3_ Quantum Dot Solar Cells. ACS Appl. Energy Mater..

[36] Lim S., Kim J., Park J.Y., Min J., Yun S., Park T., Kim Y., Choi J. (2021). Suppressed Degradation and Enhanced Performance of CsPbI_3_ Perovskite Quantum Dot Solar Cells via Engineering of Electron Transport Layers. ACS Appl. Mater. Interfaces.

[37] Shivarudraiah S.B., Ng M., Li C.H.A., Halpert J.E. (2020). All−Inorganic, Solution−Processed, Inverted Cspbi3 Quantum Dot Solar Cells with a Pce of 13.1% Achieved Via a Layer−by−Layer Fai Treatment. ACS Appl. Energy Mater..

