# Supplementary material for: Stepwise−Process−Controlled Ligand Management Strategy for Efficient and Stable Perovskite Quantum Dot Solar Cells
Source: Nanomaterials (Basel). 2023 Nov 27;13(23):3032. doi: 10.3390/nano13233032 (PMC10708130; doi:10.3390/nano13233032)
Supplement: Supplementary file 1 [file nanomaterials-13-03032-s001.zip › nanomaterials-2728966-supplementary.pdf]

Supplementary Materials

Stepwise–Process–Controlled Ligand Management Strategy for Efficient and Stable Perovskite Quantum Dot Solar Cells

Jinfei Dai [1,2,*], Wei Guo [1,2], Jie Xu [3], Ruoyao Xu [1,2], Jun Xi [1,2], Hua Dong [1,2] and Zhaoxin Wu [1,2,*]

1 Key Laboratory for Physical Electronics and Devices of the Ministry of Education School of Electronic Science and Engineering, Xi'an Jiaotong University, Xi'an 710049, China
2 Shaanxi Key Lab of Information Photonic Technique, School of Electronic Science and Engineering, Xi'an Jiaotong University, Xi'an 710049, China
3 College of Science, Xi'an University of Architecture and Technology, Xi'an 710055, China; jiexu@xauat.edu.cn
* Correspondence: daijinfei123@xjtu.edu.cn (J.D.); zhaoxinwu@mail.xjtu.edu.cn (Z.W.)

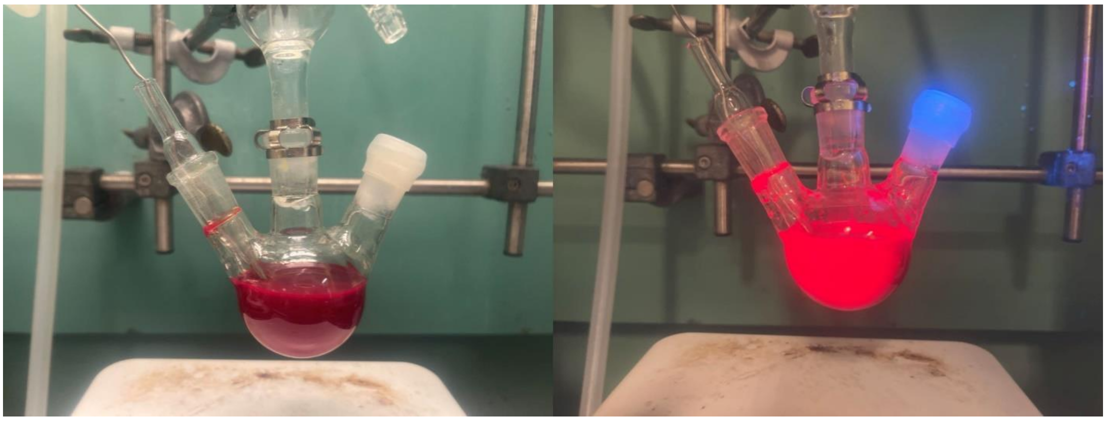

Figure S1. Photograph of as Synthesized CspbI_3 nanocrystals under sunlight (left) and UV light (right).

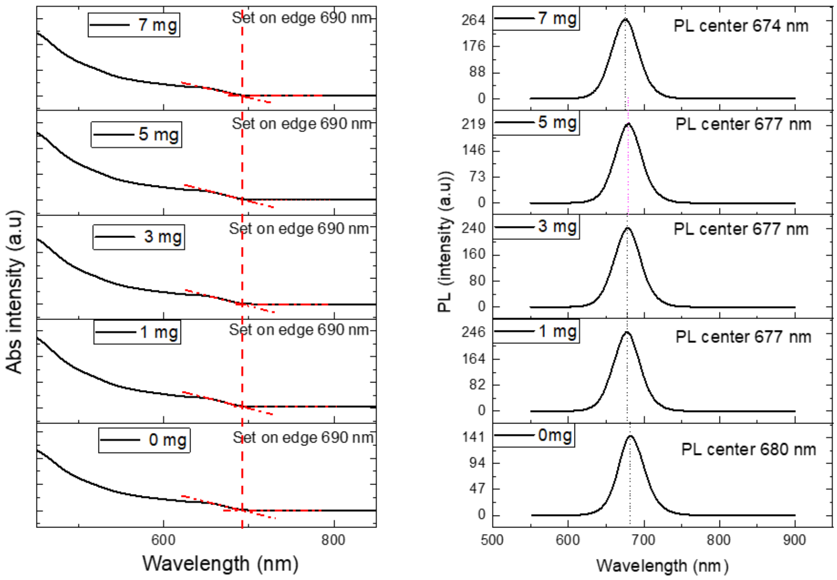

Figure S2. PL and UV-Vis absorption spectra of $CsPbI_3$ treated with different dose of BPA.

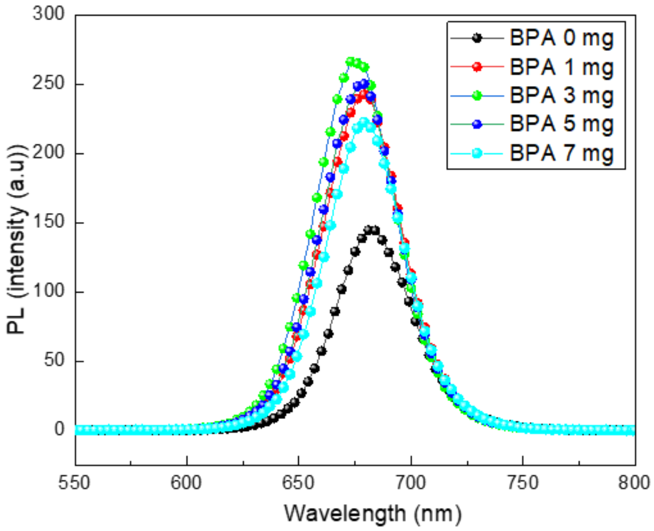

Figure S3. PL intensity of $CsPbI_3$ sample treated with different dose of BPA at the same optical density.

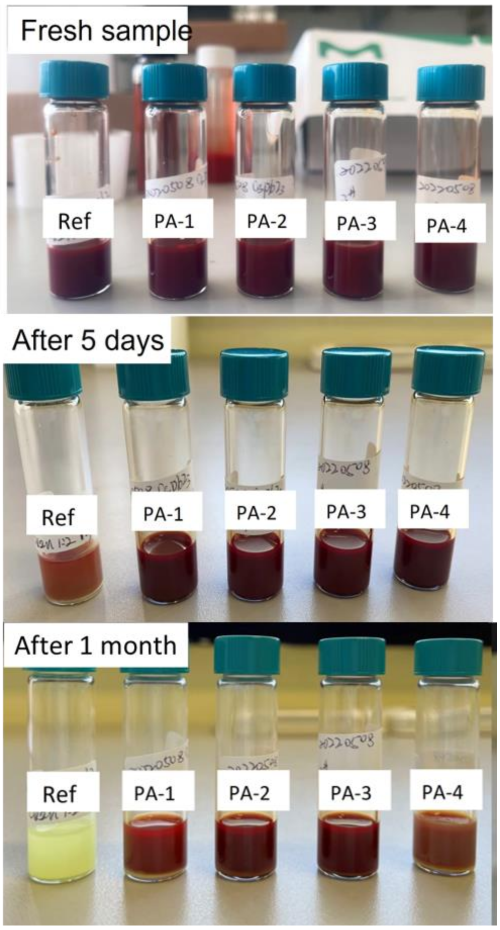

Figure S4. Photograph of $CsPbI_3$ sample treated with different dose of BPA and is varying with aging time.

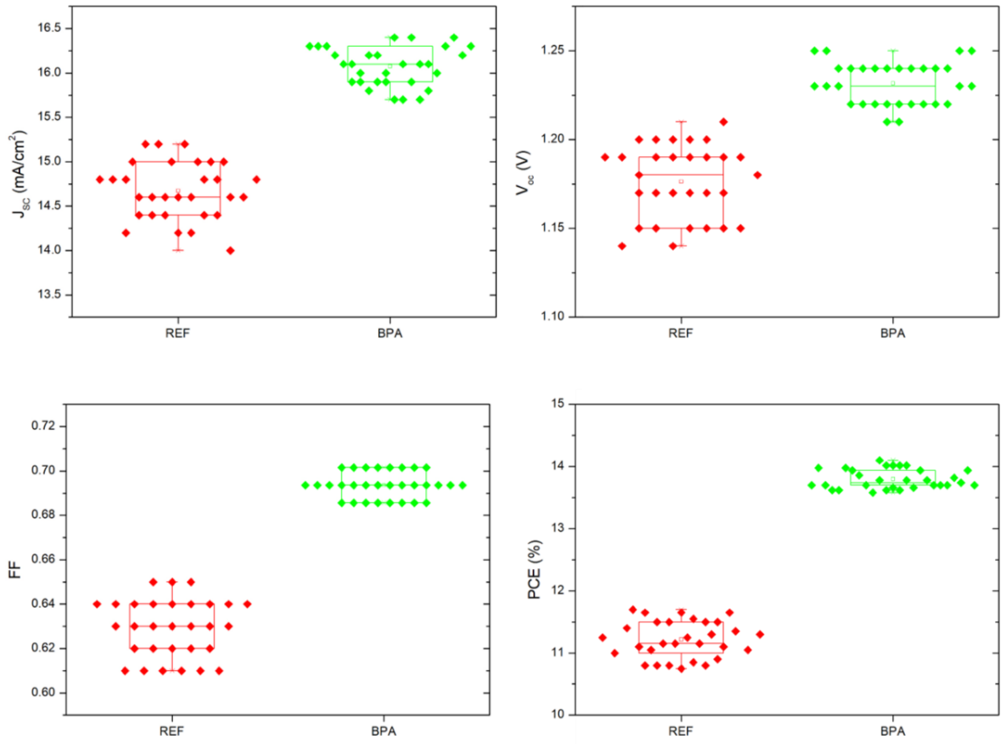

Figure S5. The statistical Photovoltaic Parameters of pristine and BPA-modified PQSCs.

Fitting Process of TRPL.

The TRPL was carried out and fitted by a biexponential decay model:

$$I_{(t)}=A_1e^{-\left(\frac{t}{\tau_1}\right)}+A_2e^{-\left(\frac{t}{\tau_2}\right)}+I_0$$

Table S1. Parameters of the time-resolved photoluminescence (TRPL) spectroscopy of control and BPA-modified perovskite films.

Samples	τ_1 (ns)	A1 (%)	τ_2 (ns)	A2 (%)	τ_{ave} (ns)
REF-$CsPbI_3$	3.98	15.25	35.19	84.75	30.42
BPA-$CsPbI_3$	4.61	11.67	56.14	88.33	50.11

Table S2. Device design strategies and performance of $CsPbI_3$ PQSCs work recently.

Device Structure	PCE [%]	Ref. [no.]
FTO/TiO_2/$CsPbI_3$ PQDs/PTB7/Ag	12.55	[1]
FTO/c-TiO_2/m-TiO_2/$CsPbI_3$ PQDs/Au	10.02	[2]
ITO/SnO_2/$CsPbI_3$ PQDs/Spiro-OMeTAD/Ag	13.66	[3]
FTO/ SnO_2/$CsPbI_3$ PQDs/Spiro-OMeTAD/MoO_x/Ag	14.5	[4]
FTO/NiO_x/$CsPbI_3$ PQDs/C_{60}/ZnO/Ag	13.10	[5]
ITO/SnO_2/$CsPbI_3$ PQDs/Spiro-OMeTAD/Ag	16.53	[6]
ITO/SnO_2/$CsPbI_3$ PQDs/Spiro-OMeTAD/ MoO_x/Ag	13.92	Our work

Table S3. The statistical Photovoltaic Parameters of pristine and BPA-modified PQSCs.

Structure	V_{OC} [V]	J_{SC} [mA/cm^2]	FF [%]	PCE [%]
REF-$CsPbI_3$	1.16 ± 0.06	14.01 ± 1.45	62.92 ± 2.53	11.03 ± 0.71
BPA-$CsPbI_3$	1.21 ± 0.03	15.98 ± 1.29	68.49 ± 2.06	13.47 ± 0.52

tDOS

Impedance spectroscopy was measured by an Electrochemical Analyzer Meter (CHI 660d). The measurement condition for thermal admittance spectroscopy (TAS) is in the dark, without bis-voltage. The trap density of states was derived from the angle frequency dependent capacitance using the equation: The energetic profile of trap density of states (tDOS) can be derived from the angular frequency dependent capacitance using the equation:

$$N_T(E_\omega) = -\frac{V_{bi}}{qW}\frac{dC}{d\omega}\frac{\omega}{k_B T}$$

where C is the capacitance, ω is the angular frequency, q is the elementary charge, k_B is the Boltzmann's constant, and T is the absolute temperature. V_{bi} and W, extracted from capacitance–voltage measurements, are the built-in potential and depletion width, respectively. The applied angular frequency ω defines an energetic demarcation,

$$E_\omega = -k_B T \ln\left(\frac{\omega_0}{\omega}\right)$$

where ω_0 is the attempt-to-escape frequency. R(CR) equivalent circuit for fitting the impedance spectra:

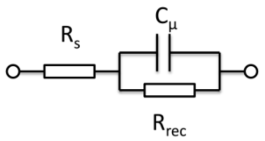

The trap states above the energy demarcation cannot contribute to the capacitance due to their slow rate to capture or emit charges.

TPV and TPC measurement

For TPV and TPC measurements, the device is connected to the 100M Ω /50 Ω input of the oscilloscope with constant background illumination provided by a white LED (Koh Hong B001). A perturbation is applied by pulsing a green LED at 530 nm (Kuang-Hong B033, Taiwan). An oscilloscope (TDS 2024B, Tektronix) recorded the resulting opto-voltage transients.

References

1. Yuan, J.; Ling, X.; Yang, D.; Li, F.; Zhou, S.; Shi, J.; Qian, Y.; Hu, J.; Sun, Y.; Yang, Y.; et al. Band-Aligned Polymeric Hole Transport Materials for Extremely Low Energy Loss α-$CsPbI_3$ Perovskite Nanocrystal Solar Cells. *Joule* **2018**, *2*, 2450.
2. Liu, Y.; Zhao, X.; Yang, Z.; Li, Q.; Wei, W.; Hu, B.; Chen, W. $Cu_{12}Sb_4S_{13}$ Quantum Dots with Ligand Exchange as Hole Transport Materials in All-Inorganic Perovskite $CsPbI_3$ Quantum Dot Solar Cells. *ACS Appl. Energy Mater.* **2020**, *3*, 3521.
3. Jia, D.L.; Chen, J.X.; Yu, M.; Liu, J.H.; Johansson, E.M.J.; Hagfeldt, A.; Zhang, X.L. Dual Passivation of CsPbI Perovskite Nanocrystals with Amino Acid Ligands for Efficient Quantum Dot Solar Cells. *Small* **2020**, *16*, 2001772.
4. Lim, S.; Kim, J.; Park, J.Y.; Min, J.; Yun, S.; Park, T.; Kim, Y.; Choi, J. Suppressed Degradation and Enhanced Performance of $CsPbI_3$ Perovskite Quantum Dot Solar Cells via Engineering of Electron Transport Layers. *ACS Appl. Mater. Interfaces* **2021**, *13*, 6119–6129.
5. Shivarudraiah, S.B.; Ng, M.; Li, C.H.A.; Halpert, J.E. All-Inorganic, Solution-Processed, Inverted Cspbi3 Quantum Dot Solar Cells with a Pce of 13.1% Achieved Via a Layer-by-Layer Fai Treatment. *ACS Appl. Energy Mater.* **2020**, *3*, 5620–5627.
6. Jia, D.L.; Chen, J.X.; Qiu, J.M.; Ma, H.L.; Yu, M.; Liu, J.H.; Zhang, X.L. Tailoring solvent-mediated ligand exchange for CsPbI perovskite quantum dot solar cells with efficiency exceeding 16.5%. *Joule* **2022**, *6*, 1632–1653.
